# Effects of sputum bacillary load and age on GeneXpert and traditional methods in pulmonary tuberculosis: a 4-year retrospective comparative study

**DOI:** 10.1186/s12879-023-08832-6

**Published:** 2023-11-27

**Authors:** Kui Li, Qianqian Hu, Jun Liu, Siyi Liu, Yingli He

**Affiliations:** 1 Department of Infectious Diseases, The First Affiliated Hospital of Xi’an Jiaotong University, 277 West YantaRoad, Xi’an, Shaanxi Province 710061 China; 2 Department of Infectious Diseases, Ankang Central Hospital, 85 South Jinzhou Road, Ankang, Shaanxi Province 725000 China; 3 Laboratory of Molecular Pathology and Tuberculosis Diseases, Ankang Central Hospital, 85 South Jinzhou Road, Ankang, Shaanxi Province 725000 China

**Keywords:** Bacillary load, Age factors, GeneXpert^®^ MTB/RIF, Tuberculosis, Comparative study

## Abstract

**Background:**

The purpose of this study was to evaluate the diagnostic value of the GeneXpert^®^ MTB/RIF (Xpert^®^), Auramine O staining method, and Lowenstein-Jensen medium for bacteriologically confirmed pulmonary tuberculosis and explore the effects of the sputum bacillary load (SBL) and qRT‒PCR threshold cycle (Ct) value on the detection methods.

**Methods:**

We retrospectively analysed the results in the Department of Infectious Disease for 49 months. The χ^2^ test was used to compare the performances of each method, receiver operating characteristic curve analysis was used to determine the optimal cut-off values, and the factors associated with a false-negative result from Xpert^®^ were analysed by logistic regression.

**Results:**

Simultaneous analysis of 980 sputum specimens showed that the positive detection rate of Xpert^®^ did not increase with increasing SBL, and there were differences between the three when SBL ≤ 1 + (all *P* < 0.05). There was a good negative correlation between the Ct value and the SBL (*P* < 0.0001). Age was an independent risk factor for false-negative Xpert^®^ results (*P* = 0.029), and when Ct < 16, the diagnostic sensitivity and specificity were both 100.00%. The optimal cut-off Ct values for resegmentation based on the drug resistance classification were < 18.6, 18.6–34.1, and > 34.1 cycles.

**Conclusions:**

Xpert^®^ was not affected by SBL but it was by age, and it is more advantageous when SBL ≤ 1 + . The results regarding rifampicin resistance were reliable, and the novel Ct segmentation was a practical and more clinically meaningful classification method for diagnosing rifampicin resistance. These findings will help improve physicians’ ability to accurately diagnose TB.

**Supplementary Information:**

The online version contains supplementary material available at 10.1186/s12879-023-08832-6.

## Background

In 2020, the World Health Organisation (WHO) reported that tuberculosis (TB) is still one of the top 10 causes of death in low- and middle-income countries, and the world’s leading cause of death from infectious diseases. It is estimated that 10 million people suffer from TB and 1.5 million die from TB each year [1]. On average, approximately 63% of the patients have a confirmed diagnosis by bacteriology, but there is still a large gap between this worldwide average and the average in high-income countries (89%) [2]. These diagnostic gaps are mainly due to the lack of highly sensitive, rapid, and available diagnostic measures. The use of better methods to ensure that people receive a timely diagnosis and the most effective treatment is of great practical significance in the prevention and control of TB and is also extremely important to public health.

The pathogenic methods of diagnosing TB include smear microscopy, culture methods, and molecular-biological examinations. *Mycobacterium tuberculosis* (*Mtb*), *nontuberculous mycobacteria* (NTM) [3, 4], *Mycobacterium leprae* [5, 6], *Nocardia* spp. [7], *Corynebacterium* spp., and *Rhodococcus equi*. [8] are known acid-fast bacillus (AFB) smear–positive bacteria. Due to the extremely low incidence of NTM in the nontropical regions of northern China [9], leprosy has been basically eliminated, and *Nocardia* spp. (hospitalisation rate 0.04/100000) [7] and *Rhodococcus equi* infection are rare, so AFB positivity is extremely important for the clinical diagnosis of TB, with a specificity as high as 98% [10], but its sensitivity is insufficient [10, 11]. Due to the slow growth of *Mtb*, the culture method not only requires professional and technical personnel and laboratories with standard biosafety levels but also has disadvantages such as lagre time demands and a poor positive isolation rate [12].

The rapid molecular biology technique GeneXpert^®^ MTB/RIF (Xpert^®^) is currently the most commonly used detection method for the rapid diagnosis of TB as recommended by the WHO [2]. Xpert^®^ can detect DNA of the *Mtb* complex and rifampicin (RIF) resistance-associated *rpoB* mutations in patient specimens, and the results can be reported in 2 h [13]. Although it has higher sensitivity than AFB and requires a lower sputum bacillary load (SBL) [14, 15], its sensitivity is still low when the lesion area is small [16]. Most of the studies on this topic were qualitative studies that included suspected pulmonary TB cases and evaluated the consistency of the drug resistance results [14–17]. The presence of confounding factors may affect the results, and quantitative analysis of the impact of SBL and threshold cycle number (Ct value) on susceptibility and resistance in confirmed cases is still lacking, so further research is urgently needed.

The purpose of this study is to evaluate the following: (1) the positive detection rates of various methods for diagnosing TB; (2) whether and how the SBL affects the positive detection rates of the three methods; (3) the risk factors affecting false-negative Xpert^®^ results; and (4) the effect of the Ct value on Xpert^®^ detection of RIF resistance.

## Methods

### Study design

We retrospectively analysed bacteriologically confirmed pulmonary tuberculosis (BC-PTB) patients who were consecutively hospitalised in Ankang Central Hospital, China. Data were collected from December 1, 2018, to December 31, 2022. Xpert^®^, the auramine O staining method (AOSM), and Lowenstein-Jensen (L-J) medium test results were the main indicators collected in this study. Data such as sex, age, SBL, and drug resistance status were also collected.

The same sputum specimens that simultaneously underwent Xpert^®^, AOSM and L-J medium examinations were included in the analysis of the effect of SBL on the positive detection rate of the different methods. All samples for which we had the results of the proportional method for drug susceptibility testing (DST) and the Xpert^®^ test were included in the evaluation of the diagnostic efficacy of RIF resistance.

### Inclusion and exclusion criteria

BC-PTB corresponded to categories A15 and A19, including all subcategories (A15.0 to A15.9, A19.0 to A19.9), according to the Tenth Revision of the International Statistical Classification of Diseases and Related Health Problems. We retrieved BC-PTB from all diagnoses of discharged patients and defined it as patients who met one of the following criteria were included [18]: (1) any positive AOSM result of sputum or body fluid specimens; (2) positive mycobacterial isolation and culture in L-J medium; and (3) a positive result from Xpert^®^.

The exclusion criteria included: (1) negative results on all three tests; (2) TB confirmed only by histology; and (3) lack of laboratory results.

Body fluid samples include bronchoalveolar lavage fluid, pleural effusion, ascites, local secretions or pus, cerebrospinal fluid and urine, etc. Sample counting rule, when the two results were positive at the same time, the sputum culture results were used as the standard, and when the results were inconsistent, the positive result was taken as the result. Specimens from the same patient were only counted once according to the highest SBL.

### Test method

The auramine O staining solution and the type II L-J culture tubes were provided by Baso Diagnostics, Inc., Zhuhai, China, and the examination was performed in a KRJ/TTR500 automatic smear staining machine (Xiangyang Courager Medical Apparatus, Xiangyang, China). A water-jacket incubator (GH6000) was provided by Tianjin Taisite Instrument Co., Ltd. The *Mtb rpoB* gene and mutation detection kit (real-time fluorescence PCR method) and its supporting equipment were obtained from Cepheid Shanghai Trading Co., Ltd. At least 1 ml, 3 ml, and 2 ml of sputum were collected for Xpert^®^, AOSM, and L-J medium tests, respectively, all of which were stored at 2–8 °C after collection and during submission, and submitted for testing within 48 h-after sampling. The AOSM and Xpert^®^ test were performed directly from the sputum, and L-J medium was used from the sediment after decontamination. The operation steps of each method are shown in Supplementary table S1. Sputum smear and culture results were reported in accordance with the *Diagnostic criteria and principles of management of infectious pulmonary tuberculosis* (see Supplementary table S2) [19].

*Mtb* test results and Ct ranges were classified as follows: “very low” (Ct > 28), “low” (Ct = 22–28), “medium” (Ct = 16–22), and “high” (Ct < 16). Any two probes with a Ct difference of > 4 cycles were reported as drug resistant. “Uncertain RIF resistance” was reported if the Ct of the first probe > 34.5 cycles or the Ct of the last probe > 38 cycles.

### Sample size

In our study, we employed a sample size estimation formula for comparing rates among multiple samples using a completely randomized design:

$$n=\frac{1641.6\uplambda }{{\left({sin}^{-1}\sqrt{{p}_{max}}{-sin}^{-1}\sqrt{{p}_{min}}\right)}^{2}} = \frac{1641.6\times 12.65}{{\left({sin}^{-1}\sqrt{0.83}-{sin}^{-1}\sqrt{0.59}\right)}^{2}}=\frac{20766.24}{{\left(-12.86\right)}^{2}}\approx 126$$

Where *n* represents the required sample size for each group, *Ƥmax* and *Ƥmin* indicate the maximum and minimum rates, respectively, and *k* is the value obtained using a table look-up method according to α, β, and degree of freedom *ν* = *k*-1. The positive rates of Xpert^®^ [20], AOSM [21] and L-J medium [22] were 83.00%, 71.85%, and 58.62% via corresponding literature retrieval, respectively. Considering that *Ƥmax* = 0.83 and *Ƥmin* ≈ 0.59 in this case, assuming α = 0.05, β = 0.10 and *ν* = 3–1 = 2, it was obtained that λ = 12.65 using the table look-up method.

Furthermore, the square root arcsine transformation is based on angular transformation. By substituting the above data into the formula, it was calculated that at least 126 cases were needed for each group, and a total of 378 cases were required for the three groups.

### Statistical analysis

The relationship between the number of positive cases found by the different methods was represented by a Venn diagram. Nonnormally distributed measurement data are expressed as the median and interquartile range, and the Wilcoxon rank-sum test was used for comparisons between groups. For count data, the Cochran-Armitage trend test was used to determine the trend of the positive detection rate with SBL changes, and the χ^2^ test and Fisher’s exact test in an R × C contingency table were used for intergroup comparisons. The false negative rate of Xpert^®^ was analysed by a backwards logistic regression model. Two variables were considered collinear if the absolute value of their correlation coefficient (|r|) was ≥ 0.5. Missing data of the independent variables were replaced by the series mean. Consistency between methods was assessed using the kappa coefficient, and receiver operating characteristic (ROC) curve analysis was used to determine the optimal cut-off value and diagnostic efficacy. SPSS 22.0 (IBM Corp., Armonk, NY, USA) and GraphPad Prism 8.0.2 (GraphPad Software, San Diego, CA, USA) were used for data analysis. *P* < 0.05 was the test level for intergroup comparisons.

## Results

### Participant characteristics

**Fig. 1:**
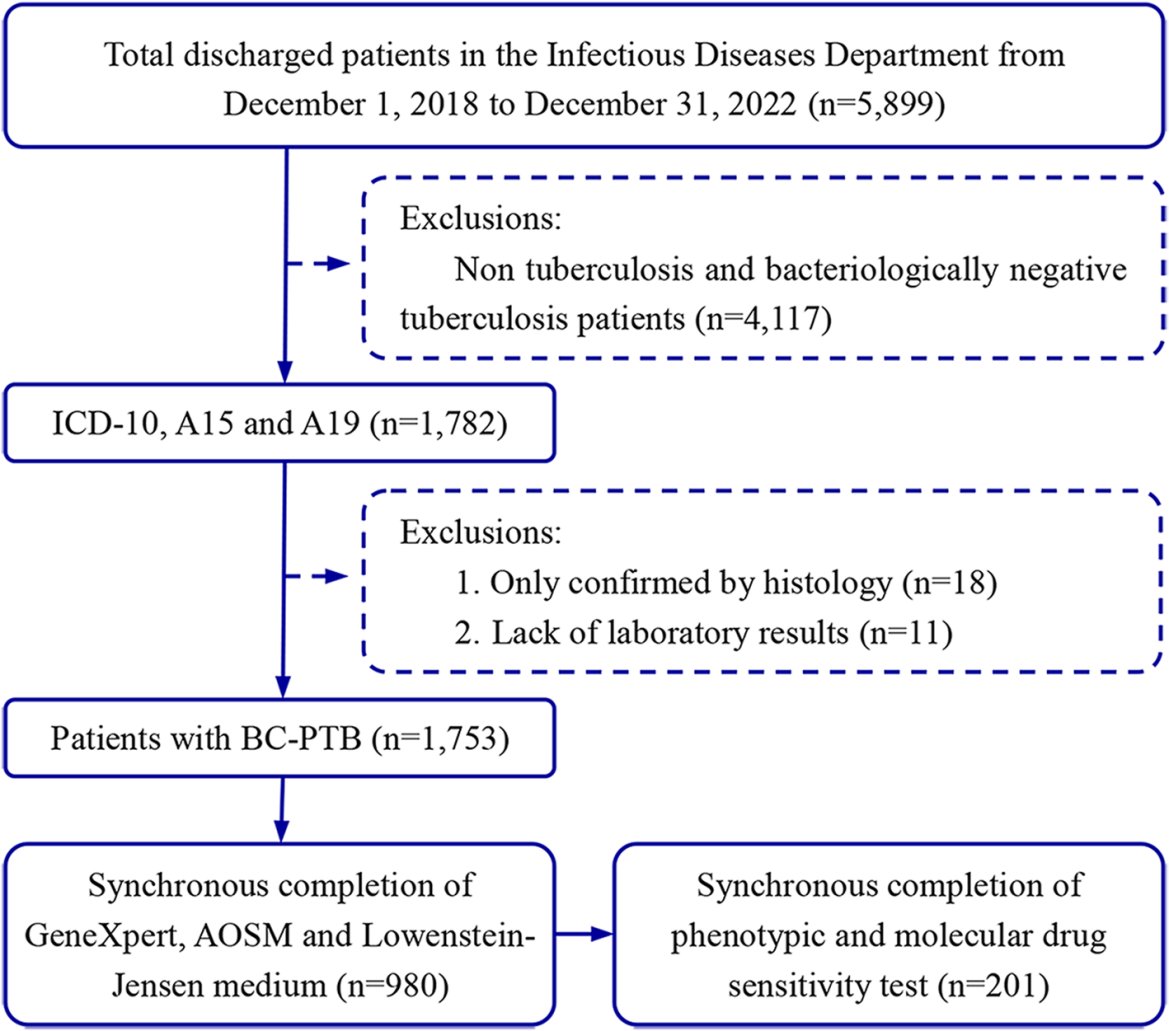
The flowchart of this study. AOSM: auramin O staining method; BC-PTB: bacteriologically confirmed pulmonary tuberculosis; ICD-10: the Tenth Revision of the International Statistical Classification of Diseases and Related Health Problems

The flowchart of this study is shown in Fig. 1. A total of 1753 cases of BC-PTB were included in the analysis. The number of cases obtained by each method was 1589, 1457, and 1089, respectively. The median patient age was 55.00 (41.00–66.00) years old. There were 1291 males (73.65%) and 462 females (26.35%), for a male to female ratio of 2.79:1. There was no significant difference in age or sex between any methods (H = 0.397, *P* = 0.820, and χ^2^ = 1.398, *P* = 0.497) (Table 1). A total of 980 sputum specimens were simultaneously detected by the three methods, and 201 cases were successfully matched and synchronously put through DST by the two methods (Fig. 1).

**Table 1:** Baseline characteristics of the entire study population (*n* = 1753)

	Xpert^®^	AOSM	L-J medium	Test value (H or χ^2^)	*P* value
Total	1589	1457	1089	-	-
Age,years, M (IQR)	55.00 (42.00–67.00)	54.00 (41.00–66.00)	54.00 (43.00–66.00)	0.397	0.820
Male, sex, n (%)	1157 (72.81)	1086 (74.54)	810 (74.38)	1.398	0.497
Retreatment^a^, n (%)	437 (27.50)	414 (28.41)	318 (29.20)	0.943	0.624
Diabetes mellitus, n (%)	164 (10.32)	177 (12.15)	134 (12.30)	3.468	0.177
Positive, n (%)	1550 (97.55)	1052 (72.20)	825 (75.76)	396.814	< 0.0001
SBL^b^, n (%)				767.353	< 0.0001
*Mtb* DNA positive	511 (32.97)	0 (0.00)	0 (00.00)	-	-
Number of colony	189 (12.19)	155 (14.73)	167 (20.24)	-	-
1+	290 (18.71)	275 (26.14)	206 (24.97)	-	-
2+	232 (14.97)	258 (24.52)	136 (16.49)	-	-
3+	166 (10.71)	191 (18.16)	148 (17.94)	-	-
4+	162 (10.45)	173 (16.45)	168 (20.36)	-	-
DST, n (%)	1589 (100.00)	-	224 (20.57)	-	-
RIF resistance	207 (13.03)	-	50 (22.32)	13.940	< 0.0001
Simultaneous DST^c^, n (%)	201 (12.65)	-	201 (89.73)	-	-
RIF resistance	49 (24.38)	-	43 (21.39)	0.507	0.476

*AOSM* Auramin O staining method, *DNA* Deoxyribonucleic acid, *DST* Drug sensitivity test, *IQR* Interquartile range, *L-J medium* Lowenstein-Jensen medium, *RIF* Rifampin, *M* Median, *Mtb* Mycobacterium tuberculosis, *SBL* Sputum bacillary load

^a^Retreatment is defined as patients who received irregular anti-TB treatment ≥ 1 month in the past, had failed initial treatment, had their sputum samples turn positive for tuberculosis, and had chronically excreted bacteria

^b^The reporting standards for sputum bacterial load can be found in Supplementary Table S2

^c^Refers to the completion of Xpert^®^ and L-J medium drug sensitivity tests by the same patient

### Positive detection rate and diagnostic efficacy of different methods

**Fig. 2:**
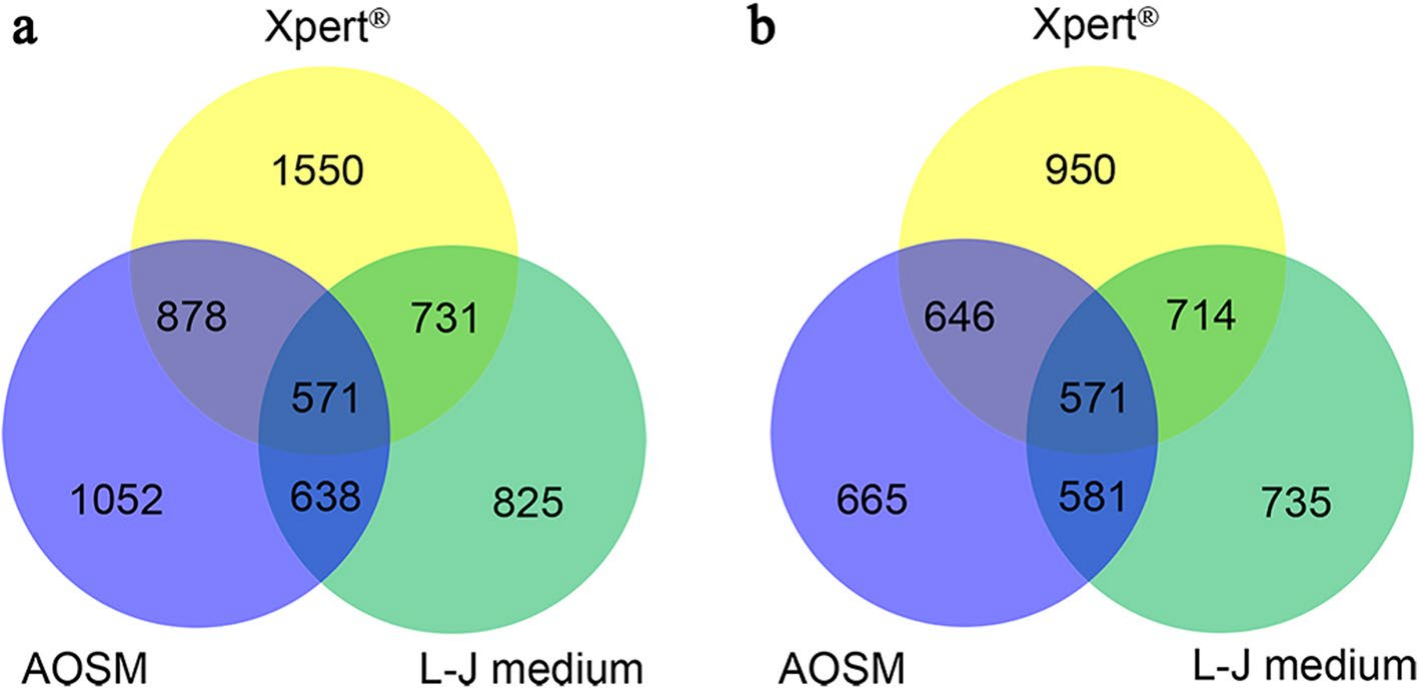
Venn diagram representing the number of positive results from the Xpert^®^, AOSM and L-J medium tests among 1753 patients (**a**) and 980 patients (**b**) with synchronous sputum detection. AOSM: auramine O staining method; L-J medium: Lowenstein-Jensen medium

The positive detection rates of Xpert^®^, AOSM and L-J medium in BC-PTB were 97.55%, 72.20%, and 75.76%, respectively (χ^2^ = 396.814, *P* < 0.0001) (Table 1). The relationship of the positive results between different methods is shown in Fig. 2a. Compared with the Xpert^®^ method, which had the highest positive detection rate, the positive detection rate of AOSM combined with L-J medium was lower, and the positive detection rate of Xpert^®^ combined with AOSM or L-J medium was higher than that of Xpert^®^ alone (all *P* < 0.05) (see Supplementary table S3). Xpert^®^ increased the positive detection rate by 16.43% in patients with negative results of sputum smears and cultures (i.e., *Mtb* DNA positive) (Table 2).

**Table 2:** Simultaneous analysis of the effect of sputum bacillary load on the three methods (*n* = 980)

Sputum bacillary load^a^	N (%)	Xpert^®^	AOSM	L-J medium	χ^2^ value	*P* value
*Mtb* DNA positive	161 (16.43)	161 (100.00)	0 (0.00)	0 (0.00)	483.000	< 0.0001
Number of colony	176 (17.96)	167 (94.89)	119 (67.61)	150 (85.23)	46.782	< 0.0001
1+	223 (22.75)	213 (95.52)	156 (69.96)	186 (83.41)	51.579	< 0.0001
2+	138 (14.08)	129 (93.48)	118 (85.51)	122 (88.41)	4.637	0.098
3+	131 (13.37)	130 (99.24)	124 (94.66)	128 (97.71)	4.769^b^	0.092
4+	151 (15.41)	150 (99.34)	148 (98.01)	149 (98.68)	1.045^b^	0.875
χ^2^ value^c^	-	0.427	342.101	328.183	-	-
*P* value	-	0.513	< 0.0001	< 0.0001	-	-

*AOSM* Auramin O staining method, *DNA* Deoxyribonucleic acid, *L-J medium* Lowenstein-Jensen medium, *Mtb* Mycobacterium tuberculosis

^a^Refers to sputum bacillary load was from low to high

^b^Fisher`s exact test

^c^Cochran Armitage trend test

Using L-J medium as the gold standard, the sensitivity of Xpert^®^ combined with AOSM was further improved over that of either single method (see Supplementary table S4).

### Effect of SBL on the positive detection rate

There were significant differences between the three methods in terms of SBL composition (*P* < 0.0001) (Table 1). The relationship between the three examination methods and the number of positive cases in the 980 samples is shown in Fig. 2b.

The positive detection rate of AOSM plus L-J medium increased with increasing SBL (horizontal items: *P* < 0.0001). However, Xpert^®^ did not show this trend (horizontal items: *P* > 0.05) (Table 2).

The results of the intergroup comparison showed that there was a significant difference between the three methods when SBL was low (≤ 1 +) (vertical items: all *P* < 0.01), As the SBL increased, this last difference gradually disappeared (vertical items: *P* > 0.05) (Table 2).

### Risk factors for a false-negative Xpert^®^ result

**Fig. 3:**
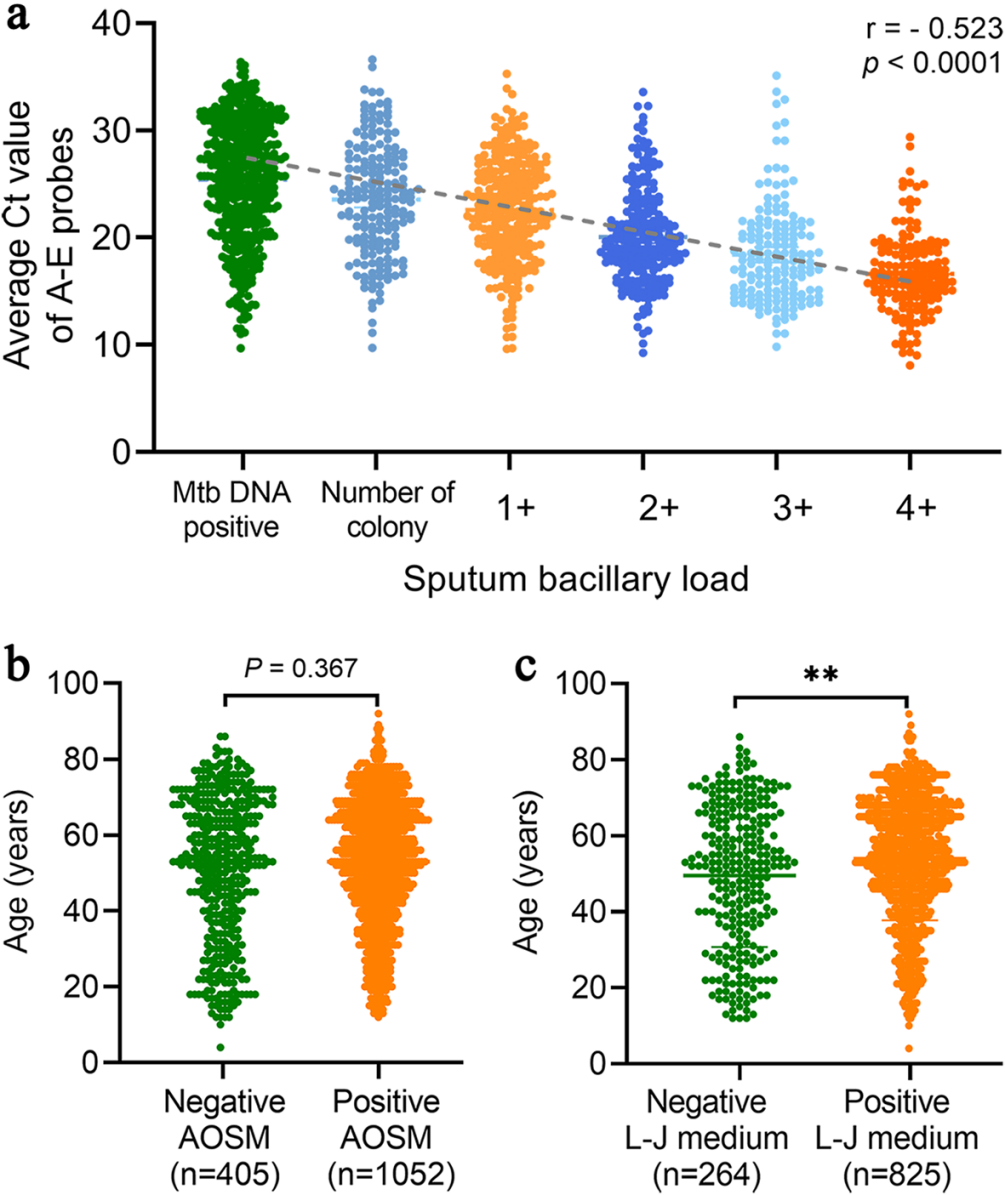
Correlation between the average Ct value of probes A-E and SBL grade (**a**), the dotted line represents the slope of the connecting median. the difference in age between AOSM (**b**) and L-J medium (**c**). AOSM: auramine O staining method; L-J medium: Lowenstein-Jensen medium, **P < 0.01

There was a good negative correlation between the average Ct value of probes A-E and the SBL grade in the 1550 cases positive for *Mtb* by the Xpert^®^ test [*r* =  − 0.523, 95% confidence interval (CI) (-0.395) − (-0.506), *P* < 0.0001] (Fig. 3a and Supplementary table S5).

Univariate screening showed there were significant differences between the age and the SBL in true positive and false-negative Xpert^®^ result (Table 3), multivariate logistic regression analysis showed that there was no serious collinearity between the factors (maximum |*r*|= 0.131), that the model was a good fit (*R* square = 0.013; Hosmer and Lemeshow test: χ^2^ = 7.592, *P* = 0.474) and that age was an independent risk factor for a false-negative Xpert^®^ result (odds ratio = 0.980, 95% CI 0.963 – 0.998, *P* = 0.029) (Table 4). There were no differences in age between the AOSM positive and negative groups (z = -0.901, *P* = 0.367) (Fig. 3b), but there were significant difference in L-J medium (z = -3.071, *P* = 0.002) (Fig. 3c).

**Table 3:** Univariate analysis of possible risk factors for false-negative Xpert^®^

Variables	True-positive Xpert^®^ (*n* = 1550)		False-negative Xpert^®^ (*n* = 39)		Test value (z or χ^2^)	*P* value
	Missing data	Observed data	Missing data	Observed data		
Age, years, M (IQR)	0 (0.00)	55.00 (42.00–67.00)	0 (0.00)	52.00 (28.00–62.00)	-1.992	**0.046**
Male, sex, n (%)	0 (0.00)	1128 (72.77)	0 (0.00)	29 (73.36)	0.048	0.826
Retreatment, n (%)	13(0.84)	424(27.35)	1 (2.56)	13 (33.33)	0.558	0.455
Diabetes mellitus, n (%)	0 (0.00)	158 (10.19)	0 (0.00)	6 (15.38)	0.618^a^	0.432
AOSM-negative, n (%)	292 (18.84)	380 (24.52)	1 (2.56)	12 (30.77)	0.033	0.856
L-J medium-negative, n (%)	573 (36.97)	246 (15.87)	9 (23.08)	9 (23.08)	0.358	0.550
SBL, n (%)	0 (0.00)	1550 (100.00)	0 (0.00)	39 (100.00)		
*Mtb* DNA positive		511 (32.97)		0 (0.00)	18.952	**< 0.0001**
Number of colony		189 (12.19)		11 (28.21)	7.469^a^	**0.006**
1+		290 (18.71)		13 (33.33)	5.272	**0.022**
2+		232 (14.97)		12 (30.77)	7.308	**0.007**
3+		166 (10.71)		2 (5.13)	0.733^a^	0.392
4+		162 (10.45)		1 (2.56)	1.786^a^	0.181

Bold represents the items entered into the multivariate analysis

*AOSM* Auramin O staining method, *IQR* Interquartile range, *L-J medium* Lowenstein-Jensen medium, *M* Median, *Mtb* Mycobacterium tuberculosis, *SBL* Sputum bacillary load

^a^Continuity correcton of Pearson`s chi-square test

**Table 4:** Logistic regression analysis of false-negative Xpert^®^

Variables	*β*-coefficient	*S*_*‾χ*_ value	*Wald χ*^2^ value	*P* value	Odds ratio	95% *CI*
Intercept	-3.018	0.514	34.499	0.000	0.049	
Age	-0.020	0.009	4.768	0.029	0.980	0.963–0.998
SBL	0.107	0.094	1.291	0.256	1.113	0.925–1.339

*CI* Confidence interval, *SBL* Sputum bacillary load

### Comparison of Xpert^®^ and the proportional method for DST in detecting RIF resistance

Among the 207 cases with *rpoB* mutations, 55.56% and 21.74% were located in probe E and probe D, respectively (see Supplementary table S6).

Among the 201 cases simultaneously detected by the two methods, comparison of the Xpert^®^ and proportional methods for DST showed that the Youden index, sensitivity, specificity, positive predictive value, and negative predictive value in detecting rifampicin-resistant tuberculosis (RR-TB) were 90.28%, 95.34%, 94.94%, 83.67%, and 98.68%, respectively (Table 5), with no significant difference between the two methods (χ^2^ = 0.507, *P* = 0.476). The kappa coefficient was 0.859 (95% CI 0.775–0.943, *P* < 0.0001).

**Table 5:** Comparison of Xpert^®^ and L-J medium on the results and Ct value stratified in detecting RIF resistance

Xpert^®^	Outcomes	Proportional DST of L-J medium, n		Total	Sensitivity (%)	Specificity (%)	PPV (%)	NPV (%)
		Resistant	Sensitive					
Test results, *n* = 201^a^	Resistant	41	8	49				
	Sensitive	2	150	152	95.34	94.94	83.67	98.68
Stratified according to Ct value, *n* = 194								
Low (Ct > 22)^b^, *n* = 44	Resistant	11	3	14				
	Sensitive	0	30	30	100.00	90.91	78.57	100.00
Medium (Ct 16–22), *n* = 94	Resistant	23	5	28				
	Sensitive	1	65	66	95.83	92.86	82.14	98.48
High (Ct < 16), *n* = 56	Resistant	7	0	7				
	Sensitive	0	49	49	100.00	100.00	100.00	100.00

*Ct* Threshold cycle, *DST* Drug susceptibility testing, *L-J medium* Lowenstein-Jensen medium, *N* Number, *NPV* Negative predictive value, *PPV* Positive predictive value

^a^Mycobacterium tuberculosis was not detected by Xpert^®^ in seven cases

^b^There were only seven cases in the Ct > 28 group. Due to the small number of cases, the cases were merged into the adjacent Ct = 22–28, which were identified as Ct > 22

### Effect of the Ct value on the results and efficacy of Xpert^®^ in detecting RIF resistance

The detection of RIF resistance by Xpert^®^ increased with decreasing Ct (χ^2^ = 8.229, *P* = 0.004). RIF resistance uncertainty by Xpert^®^ detection was statistically significant between Ct > 28 cycles and other levels (χ^2^ = 43.159, *P* < 0.0001) (see Supplementary table S7 and fig. S1).

Using L-J medium as the gold standard, the sensitivities of Xpert^®^ in the detection of RIF resistance at Ct > 22, Ct = 16–22, and Ct < 16 were 100.00%, 95.83%, and 100.00%, respectively, and the specificities were 90.91%, 92.86%, and 100.00% (Table 5).

### Exploration of resegmentation of Ct values based on clinical drug resistance classification

The optimal cut-off of the average Ct value between RIF resistance and sensitivity of the five probes was 18.6, the area under the ROC curve was 0.775 (95% CI 0.741–0.808, *P* < 0.0001), the sensitivity was 74.21%, and the specificity was 66.18%. The optimal cut-off of the Ct value between drug resistance uncertainty and sensitivity was 34.1, the area under the ROC curve was 0.933 (95% CI 0.868–0.997, *P* < 0.0001), the sensitivity was 76.47%, and the specificity was 99.85% (Tables 6 and 7 and see Supplementary fig. S2).

**Table 6:** Changes in Ct values between RIF resistance and sensitivity (*n* = 1550)

Xpert^®^ test results	N	Ct of rpo B mutations, median (IQR)					Probes of A-E, median (IQR)
		Probe A	Probe B	Probe C	Probe D	Probe E	
RIF-resistant	207	18.60 (14.70–23.20)	20.80 (17.30–25.20)	20.00 (16.70–24.70)	19.40 (13.90–24.20)	0.00 (0.00–22.20)	16.66 (13.68–20.20)
RIF-sensitive	1326	21.70 (17.60–26.40)	22.80 (18.90–27.10)	21.90 (17.80–26.50)	22.80 (18.70–27.40)	23.30 (19.38–28.10)	22.52 (18.48–27.06)
Susceptibility to RIF “indeterminate”	17	35.00 (33.95–35.60)	34.70 (33.55–35.50)	34.20 (33.25–35.10)	35.70 (35.15–36.75)	36.90 (18.15–37.85)	35.10 (31.42–35.90)

*Ct* Threshold cycle, *IQR* Interquartile range, *RIF* Rifampin

**Table 7:** ROC analysis of Ct value between rifampicin resistance and sensitivity

	AUC(95% CI)	*P* value	Youden index (%)	Cutoff value	Sensitivity (%)	Specificity (%)
RIF-resistant Vs. RIF-sensitive	0.775 (0.741–0.808)	< 0.0001	39.39	18.6	74.21	66.18
Susceptibility to RIF “indeterminate” Vs. RIF-sensitive	0.933 (0.868–0.997)	< 0.0001	76.32	34.1	76.47	99.85

*AUC* Area under the curve, *CI* Confidence interval, *Ct* Threshold cycle, *RIF* Rifampin, *ROC* Receiver operating characteristic curve

## Discussion

In this study, the diagnostic value of each method was evaluated in depth through a comparative analysis of the results of the three detection methods for BC-PTB, and the trends and key aspects of the effect of SBL on the different methods were found, as was the effect of the Ct value on Xpert^®^ detection of RIF resistance.

In this study, 72.20% of patients with BC-PTB were positive by the AOSM, which is lower than the findings in the report of Marais et al. [23] but similar to the results from Gizaw et al. [24]. The reason for the lower findings may be related to the fact that some cases included in this study were confirmed only by DNA testing. Based on our results, it can be deduced that the main potential improvement in diagnosing TB that can be made in the region is 72.20% minus the average positive detection rate of the AOSM. The positive detection rate of Xpert^®^ was 97.55%, which was significantly higher than that of the AOSM and L-J medium. When Xpert^®^ was combined with AOSM, the positive detection rate was further improved and was superior in terms of sensitivity (98.50%) to that (95.00%) reported by Cowan et al. for repeat detection [25], but specificity was lost. The significance of this specificity loss in clinical decision-making will be limited because there are very few bacteria other than *Mtb* that are positive in the AOSM. Based on cost-effectiveness considerations, the results of this study suggest Xpert^®^ combined with AOSM as a more reasonable option than repeat Xpert^®^.

We further found that Xpert^®^ detected negative results in 16.43% of patient sputum smears and cultures (i.e., *Mtb* DNA positive) for BC-PTB. This phenomenon cannot be sufficiently explained by a negative culture due to excessive decontamination or the loss of the reproductive ability of *Mtb* after anti-TB treatment. This view is supported by the fact that there was no difference in the positive rate of Xpert^®^ between initial and retreated tuberculosis (see Supplementary table S8). Another interesting finding was that when the SBL was low (≤ 1 +), the positive detection rate of Xpert^®^ was higher than that of AOSM and L-J medium, and Xpert^®^ was more specific than reported by Dorman et al. [15]. In particular, this assay may have the greatest detection advantage in patients with negative sputum smears and negative cultures. The positive detection rate of Xpert^®^ did not increase with increasing SBL level, which may be related to the inherent limit of detection (LOD) of *Mtb* associated with each. It is common for AOSM to have a high LOD relative to culture, both observed at the whole organism and at the macro/microscopic level. On the other hand, Xpert^®^ is a molecular method with an LOD much closer to that of L-J medium culture than to that of AOSM. These points are directly demonstrated by Supplementary table S9 (Xpert^®^ basically confirming 92.86% of the TB patients with negative sputum smears and positive cultures), in line with related reports [26–28].

A disadvantage is that microscopy and Xpert^®^ cannot distinguish viable from nonviable *Mtb*, which might act as a confounder in interpreting the results (see Supplementary table S9, where a high proportion of smear-positive, culture-negative specimens is shown to be confirmed by Xpert^®^). Although Xpert^®^ Ultra improves the sensitivity to low SBL [29, 30], The increase in sensitivity of Xpert^®^ ultra is at the cost of reducing specificity [31], which was possibly attributed to the increased detection rate of non-viable *Mtb*, resulting in false positive results partially [32, 33]. There was a relatively higher specificity of Xpert^®^ in human immunodeficiency virus-positive patients or patients with a medical history of tuberculosis; but a lower proportion of “uncertain” RIF detection results [34–36], showing stronger accessibility at this stage. It may provide a potential reference for medical decision-making for cases with a medical history of tuberculosis in a high-burden setting and with Ultra trace results via Xpert^®^ ultra test.

We must be aware of the effect of false positives on the results [15]. The following can help to distinguish between true and false positives: (1) Patients diagnosed for the first time. (2) Patients with symptoms of tuberculosis poisoning. (3) Patients with progressive pulmonary lesions were dynamically observed. (4) Patients who do not respond to regular antibiotic treatment. In this study, 2.45% of the patients still showed false-negative Xpert^®^ results. The possible reasons are that (1) excessive alkali treatment during specimen preparation resulted in the destruction of *Mtb* DNA and (2) patient age was negatively associated with false-negative Xpert^®^ results, with more false negative results reported for younger patients. This was unrelated to the SBL but is probably a result of he lighter lung lesions in younger patients, similar findings have been made in L-J medium method. In recent years, multiple cross-displacement amplification coupled with nanoparticle-based lateral flow device technology has shown high sensitivity and high specificity [37], but large-scale clinical data and experimental data are lacking.

In our study, Xpert^®^ and L-J medium showed differences in the detection rate of RIF resistance, but this difference disappeared after simultaneous sputum detection and analysis (Table 1), which may be related to specimen selection bias (for patients with long hospital stays, poor outcomes, and a history of treatment, clinicians prefer DST after sputum cultures, whereas the initial examination by Xpert^®^ may include more new patients). The RIF resistance rate was lower than that of Su et al. (28.70%) [38], which may be related to the differences in regional economic development and in the availability of drug resistance testing methods. However, the results of the two tests for RIF resistance were highly consistent. In addition, the proportion of multidrug-resistant tuberculosis among RR-TB is highly consistent with a WHO report [78.00% (39/50) vs. 78.00%], and the proportion of preextensively resistant tuberculosis among multidrug-resistant tuberculosis was higher than that reported by the WHO [28.21% (11/39) vs. 14.99%] [2]. The difference in the previous use of quinolones in different regions may be the reason for the inconsistency between the two.

In this study, 17 cases of Xpert^®^ detection of drug resistance were uncertain, all of them in the Ct > 28 patient group, suggesting that the test results of drug resistance uncertainty were mainly from this type of patient. Observations of all case data showed that the delayed binding of probe E to codon 531 may be the main reason for the drug resistance uncertainty (see Supplementary table S10). This is in line with the reports of Ocheretina et al. and Qin et al. [39, 40], and it may be related to *rpoB* silencing mutations or the instability of the heterozygous probe/wild-type target during the experiment [41, 42]. Unfortunately, in this study, there was a lack of L-J medium control data concerning the uncertain results of these Xpert^®^ tests for RIF resistance. Among them, 8 cases were detected to have RIF resistance via Xpert^®^ test but were reported to be sensitive to RIF by L-J medium DST test, which may be related to the rpoB gene carrying “controversial” mutations (L430P, D435Y, H445C/L/N/S, L452P and I491F) [43, 44], recessive mutations [44], or inconsistent determination of critical concentrations [45, 46]. But these were all referenced by the Bactec 960 MGIT method. The detection sensitivity of MGIT can be improved by reducing the critical concentration and extending the culture time [43, 45], while the conventional L-J medium method hardly missed any mutations [47, 48]. In addition, 2 cases were resistant to RIF via Xpert^®^, but sensitive to RIF using the L-J medium method, which may be attributed to the reason that the mutation sites were located outside the rifampin resistance-determining region (RRDR) [46]. Studies based on larger sample sizes are needed for further confirmation in the future. In the above cases, the test should be repeated, and the chemotherapy regimen should be adjusted according to the traditional DST results. Taking L-J medium as the standard, when Ct was < 16, the sensitivity and specificity of the diagnosis of RIF resistance were both 100.00%. It may be an ideal method under such circumstances. In clinical practice, physicians should evaluate RIF resistance results based on the Ct value of Xpert^®^.

The Ct value classification of the Cepheid Company may be more derived from the level of PCR technology. In this study, the calculation method of the average Ct value of the five probes was not necessarily consistent with the calculation algorithm built in the system (i.e., corporate intellectual property). The recommendation to resegment Ct values based on drug resistance classification may be more clinically meaningful, but similar conclusions have not been reached previously.

This study still has the following limitations: (1) It lacked data on the clinical condition, and different clinical conditions of the study population may lead to differences in the results—for example, the impact of major clinical symptoms and signs. (2) The obtained number of patients in the Ct > 28 subgroup for analysis of RIF resistance was low (seven). The consistency results could be better supported with more follow-up data. (3) Results from the proportional method for DST were missing in 17 patients with an RIF resistance result of uncertainty by Xpert^®^. (4) In the false-negative Xpert^®^ analysis, there were many missing data for L-J medium, and the results may be inaccurate. Nevertheless, (1) the study subjects were all BC-PTB patients, and simultaneous analysis of sputum specimens was performed, which effectively avoided the influence of suspected pulmonary TB patients on the results. Indicators such as the positive detection rate can be an important reference in setting realistic targets for TB diagnosis. (2) This study is the first to report that SBL affected the key breakpoints and the risk factors for false-negative Xpert^®^, as well as the evaluation of the reliability of RIF resistance detection based on the Ct value, A suggestion was made to resegment the Ct value.

## Conclusions

The study results showed that Xpert^®^ results are not affected by SBL level (but they were by age) and are more advantageous for patients with SBL ≤ 1 + , especially in sputum smear-negative and culture-negative patients. The uncertainty of RIF resistance at high Ct values in the Xpert^®^ test should be confirmed on the basis of DST after culture, and the rate of RIF resistance increases with decreasing Ct values and is in strong agreement with the culture results. Evaluation by Xpert^®^ of RIF resistance based on sample Ct values may be the ideal approach at this stage. Resegmentation of Ct values in combination with drug resistance classification is more clinically logical and can better guide clinical treatment. These results can help improve the ability of clinicians to analyse TB diagnostic results.

### Supplementary Information

**Additional file 1: Supplementary table S1.** Operation steps of detection method. **Supplementary table S2.** Sputum bacillary load report standard. **Supplementary table S3.** Comparison of the positive detection rates between combination of different methods with Xpert^®^. **Supplementary table S4.** Comparison of three methods in the diagnosis of bacteriologically confirmed pulmonary TB. **Supplementary table S5.** Correlation between sputum bacillary load and Ct value of GeneXpert^®^. **Supplementary table S6.** The distribution of rpoB mutations detected by Xpert^®^ (*n* = 207). **Supplementary table S7.** Detection of Ct level and RIF resistance in Mtb-positive clinical specimens using Xpert^®^. **Supplementary table S8.** Comparison of the positive rate of Xpert^®^ between initial treatment and retreatment. **Supplementary table S9.** Positive detection rate of Xpert^®^ in different bacterial status of sputum (*n* = 980). **Supplementary table S10.** Clinical and laboratory characteristics of 17 cases with “indeterminate” result for susceptibility to RIF. **Supplementary figure S1.** Bi-directional influence of Ct value on Xpert^®^ detection results. **Supplementary figure S2.** Receiver operating characteristic curve analysis of Ct value for distinguishing RIF resistance detection.

## Data Availability

The datasets used and/or analyzed during the current study are available from the corresponding author on reasonable request.

## Abbreviations

AFB: Acid-fast bacillus
AOSM: Auramin O staining method
BC-PTB: Bacteriologically-confirmed pulmonary tuberculosis
Ct: Threshold cycle
DST: Drug susceptibility testing
L-J medium: Löwenstein-Jensen medium
*Mtb*: Mycobacterium tuberculosis
NTM: Nontuberculosis mycobacteria
PCR: Mycobacterium tuberculosis-specific polymerase chain reaction
RIF: Rifampicin
ROC: Receiver operating characteristic curve
RR-TB: Rifampicin-resistant tuberculosis
SBL: Sputum bacillary load
TB: Tuberculosis
Xpert^®^: GeneXpert^®^ MTB/RIF
