# Supplementary material for: Effects of sputum bacillary load and age on GeneXpert and traditional methods in pulmonary tuberculosis: a 4-year retrospective comparative study
Source: BMC Infect Dis. 2023 Nov 27;23:831. doi: 10.1186/s12879-023-08832-6 (PMC10680317; doi:10.1186/s12879-023-08832-6)

**Supplementary Material**

**Supplementary table S1:** Operation steps of detection method

**Supplementary table S2:** Sputum bacillary load report standard

**Supplementary table S3:** Comparison of the positive detection rates between combination of different methods with Xpert^®^

**Supplementary table S4:** Comparison of three methods in the diagnosis of bacteriologically confirmed pulmonary TB

**Supplementary table S5:** Correlation between sputum bacillary load and Ct value of GeneXpert^®^

**Supplementary table S6:** The distribution of rpoB mutations detected by Xpert^®^ (n = 207)

**Supplementary table S7:** Detection of Ct level and RIF resistance in Mtb-positive clinical specimens using Xpert^®^

**Supplementary table S8:** Comparison of the positive rate of Xpert^®^ between initial treatment and retreatment

**Supplementary table S9:** Positive detection rate of Xpert^®^ in different bacterial status of sputum (n = 980)

**Supplementary table S10:** Clinical and laboratory characteristics of 17 cases with “indeterminate” result for susceptibility to RIF

**Supplementary figure S1:** Bi-directional influence of Ct value on Xpert^®^ detection results

**Supplementary figure S2:** Receiver operating characteristic curve analysis of Ct value for distinguishing RIF resistance detection

| **Supplementary table S1.** Operation steps of detection method |
| --- |
| **Auramine O staining method**  (1) The smear was fixed, and auramine O staining solution was added dropwise to the stain for 15 minutes, followed by washing with water.  (2) The stain was decolorized with acidic alcohol solution for 1-2 minutes, washed with water, and decolorized and washed with water again.  (3) The stain was restained with restaining solution (potassium permanganate) for 2 to 4 minutes and washed with water.  (4) After drying, microscopic examination was performed under an LED fluorescence microscope (SDPTOP EX30, Ningbo Sunny Instruments Co., Ltd., Ningbo, China) in a dark room. A 10× ocular lens and 20× objective lens were used for microscopic examination. When a fluorescent rod-shaped substance suspected to be an acid-fast bacillus was found, the 40× objective lens was used for confirmation. |
| **L-J medium**  (1) We added 1-2 volumes of 4% NaOH to the sample and homogenized the sample by shaking.  (2) We took 0.1-0.3 ml of specimen and inoculated it aseptically on the slant of culture medium, two tubes per specimen.  (3) Inoculated culture tubes were placed in 35-37 °C incubator with 5% to 10% CO2 for incubation to promote the growth of mycobacteria. The tubes were left loosely capped at an angle for 24 hours, and then the caps were tightened and the tubes were placed vertically.  (4) The growth of the colonies was observed on day 3 and day 5 after inoculation, they were observed once a week. Colony growth and contamination were recorded. No colony growth after 8 weeks of incubation was reported as "culture negative" for mycobacterium. |
| **Xpert^®^ assay**  (1) We added the sputum specimen to a 50-ml pretreatment tube with a screw cap.  (2) We added 1-2 volumes of 4% NaOH to the pretreatment tube.  (3) We tightened the screw cap of the treatment tube and shook it on a vortex shaker for approximately 1 minute until the sputum specimen was fully liquefied.  (4) We put the pretreatment tube in a biological safety cabinet and left it at room temperature for 15 minutes.  (5) We added 45 ml of 67 mM phosphate/water buffer to the liquefied sputum.  (6) We centrifuged the tube at 3000g for 20 minutes.  (7) We removed the supernatant and added 2 ml of phosphate/water buffer.  (8) We labelled the sample number on the side wall of the Xpert® detection cassette.  (9) We used a sterile pipette to take 0.5 ml of sputum sediment and suspended it in a conical test tube with a screw cap.  (10) Using a sterile pipette, we added 1.5 ml of sample treatment reagent to 0.5 ml of sputum sediment sample, then shook this sample vigorously 10 to 20 times.  (11) After the sample stood at room temperature for at least 15 minutes, we again shook it vigorously 10 to 20 times.  (12) Confirming that the fully liquefied sample was above the lowest mark of the pipette, we added it slowly to the sample well of the detection cassette.  (13) We closed the lid of the detection cassette and put it through the automatic detection program within 30 minutes. |

| **Supplementary table S2.** Sputum bacillary load report standard^a^ |
| --- |
| Sputum smear |
| +: 1–9 bacteria/50 fields; |
| 1+: 10–49 bacteria/50 fields; |
| 2+: 1–9 bacteria/field; |
| 3+: 10–90 bacteria/field; |
| 4+: ≥ 100 bacteria/field. |
| At least 50 fields were observed for the 2+ reports and at least 20 fields were observed for 3+ and above results. |
| Lowenstein-Jensen medium |
| +: the actual colony count was reported, as the bacterial colony growth was less than 1/4 of the slope surface area; |
| 1+: bacterial colony growth accounted for 1/4 of the slope surface area; |
| 2+: bacterial colony growth accounted for 1/2 of the slope surface area; |
| 3+: bacterial colony growth accounted for 3/4 of the slope surface area; |
| 4+: bacterial colony growth accounted for entire the slope surface area. |
| ^a^ Refers to *Diagnostic Criteria and Principles of Management of Infectious Pulmonary Tuberculosis*（GB15987-1995） |

| **Supplementary table S3.** Comparison of the positive detection rates between combination of different methods with Xpert^®^ | | | | |
| --- | --- | --- | --- | --- |
| Diagnostic tools | N | Positive, n (%) | χ^2^ value | *P* value |
| Xpert**^®^** | 1589 | 1550 (97.55) |  |  |
| AOSM + L-J medium | 1059^a^ | 898 (84.80) | 147.919 | ＜0.0001 |
| Xpert**^®^** + AOSM | 1296^a^ | 1284 (99.07) | 9.603 | 0.002 |
| Xpert**^®^** + L-J medium | 1007^a^ | 998 (99.11) | 8.272 | 0.004 |
| AOSM: auramin O staining method; L-J medium: Lowenstein-Jensen medium; N: number  ^a^ The number of cases submitted to both checks synchronously. | | | | |

| **Supplementary table S4.** Comparison of three methods in the diagnosis of bacteriologically confirmed pulmonary TB | | | | | | | | |  |
| --- | --- | --- | --- | --- | --- | --- | --- | --- | --- |
| Diagnostic tools | Outcomes | L-J medium as gold standard | | Total | Sensitivity  (%) | Specificity  (%) | PPV  (%) | NPV  (%) | |
|  |  | Positive | Negative |  |  |  |  |  |  |
| AOSM (*N* = 1059^a^) | Positive | 638 | 93 | 731 |  |  |  |  | |
|  | Negative | 167 | 161 | 328 | 79.25 | 63.39 | 87.28 | 49.09 | |
| Xpert**^®^** (*N* = 1007^a^) | Positive | 731 | 246 | 977 |  |  |  |  | |
|  | Negative | 21 | 9 | 30 | 97.21 | 3.53 | 74.82 | 30.00 | |
| AOSM + Xpert**^®^** (*N* = 980^a^) | Positive | 724 | 245 | 969 |  |  |  |  | |
|  | Negative | 11 | 0 | 11 | 98.50 | - | 74.72 | - | |
| AOSM: auramin O staining method; L-J medium: Lowenstein-Jensen medium; N: number; NPV: negative predictive value; PPV: positive predictive value  ^a^ Number of cases completed simultaneously with the gold standard. | | | | | | | | | |

| **Supplementary table S5.** Correlation between sputum bacillary load and Ct value of GeneXpert^®^ | |
| --- | --- |
| Sputum bacillary load, n (%) | Ct of rpo B probes （A-E）, median (IQR) |
| *Mtb* DNA positive，511 (32.97) | 26.50（21.80-30.30） |
| Number of colony，189 (12.19) | 24.20（20.50-27.60） |
| 1+，290 (18.71) | 23.20（19.80-26.33） |
| 2+，232 (14.97) | 19.80（17.60-22.78） |
| 3+，166 (10.71) | 18.30（15.68-20.90） |
| 4+，162 (10.45) | 16.60（14.88-19.13） |
| Ct: threshold cycle; DNA: deoxyribonucleic acid; IQR: interquartile range; Mtb: mycobacterium tuberculosis | |

| **Supplementary table S6.** The distribution of rpoB mutations detected by Xpert^®^ (n = 207) | | |
| --- | --- | --- |
| Molecular probes | Possible main  mutation sites | rpo B mutattion,  *N* (%) |
| Probe A | L511P | 36 (17.39) |
| Probe B | D516V | 16 (7.73) |
| Probe C | S522L | 5 (2.42) |
| Probe D | H526Y, H526D | 45 (21.74) |
| Probe E | S531L, S531W | 115 (55.56) |
| Probes A+B | L511P+ D516V | 9 (4.35) |
| Probes A+D | L511P+H526Y or H526D | 1 (0.48) |
| If none of the five probes has a Ct value of 0 and any two probes have a Ct value > 4, the probe with the lowest Ct value was used for counting. There were a total of 11 cases of this condition, including 5 cases of Probe A, 2 case of Probe B, 3 cases of Probe C and 1 cases of Probe D | | |

| **Supplementary table S7.** Detection of Ct level and RIF resistance in Mtb-positive clinical specimens using Xpert^®^ | | | | |
| --- | --- | --- | --- | --- |
| *Mtb* detection level | *N* | RIF-resistant, n (%) | RIF-sensitive | Susceptibility to RIF “indeterminate” , n (%) |
| Very low（Ct > 28） | 268 | 31（11.57） | 220 | 17（6.34） |
| Low（Ct 22-28） | 463 | 44（9.50） | 419 | 0 |
| Medium（Ct 16-22） | 574 | 90（15.68） | 484 | 0 |
| High（Ct＜16） | 245 | 42（17.14） | 203 | 0 |
| χ^2^ value |  | 8.229^a^ |  | 43.159 |
| *P* value |  | 0.004 |  | ＜0.0001 |
| Ct: threshold cycle; Mtb: mycobacterium tuberculosis; RIF: rifampin  ^a^ Cochran Armitage trend test. | | | | |

| **Supplementary table S8.** Comparison of the positive rate of Xpert^®^ between initial treatment and retreatment | | | |
| --- | --- | --- | --- |
| **Therapeutic Category** | **The positive detection rate of Xpert^®^, N (%)** | *χ*^2^ value | *P* value |
| Initial treatment^a^ (n = 1251) | 1113 (88.97) |  |  |
| Retreatment (n = 485) | 424 (87.42) | 0.823 | 0.364 |
| TB: tuberculosis disease  ^a^ Initial treatment are defined as not starting anti-TB treatment or being on anti-TB treatment for <1 month. | | | |

| **Supplementary table S9.** Positive detection rate of Xpert^®^ in different bacterial status of sputum (n = 980) | |
| --- | --- |
| **Bacterial status of sputum** | **The positive detection rate of Xpert^®^, N (%)** |
| Smear-negative, culture-negative TB, n = 161 | 161 (100.00) |
| Smear-negative, culture-positive TB, n = 154 | 143 (92.86) |
| Smear-positive, culture-negative TB, n = 84 | 75 (89.29) |
| Smear-positive, culture-positive TB, n = 581 | 571 (98.28) |
| TB: tuberculosis disease | |

| **Supplementary table S10.** Clinical and laboratory characteristics of 17 cases with “indeterminate” result for susceptibility to RIF | | | | | | | | |
| --- | --- | --- | --- | --- | --- | --- | --- | --- |
| Patient | Sample type | SBL | Ct of rpo B probes | | | | | Max ΔCt |
|  |  |  | A | B | C | D | E |  |
| 1 | Sputum | The number of colony | 35.4 | 34.7 | 34.2 | 35.6 | 0.0 | > 30 |
| 2 | BALF | DNA positive | 34.0 | 33.6 | ↓33.4 | 34.8 | 36.3↑ | 2.9 |
| 3 | Sputum | 3+ | 34.9 | 33.8 | ↓33.2 | 35.9 | 37.7↑ | 4.5 |
| 4 | Sputum | DNA positive | 35.3 | 34.7 | ↓33.5 | 38.3↑ | 37.7 | 4.8 |
| 5 | BALF | DNA positive | 33.9 | ↓32.9 | 33.4 | 35.5 | 36.4↑ | 3.5 |
| 6 | Sputum | DNA positive | 34.6 | 33.5 | ↓33.1 | 35.6 | 38.3↑ | 5.2 |
| 7 | Sputum | DNA positive | 35.0 | 34.0 | ↓33.3 | 35.7 | 38.0↑ | 4.7 |
| 8 | Sputum | DNA positive | 33.7 | 33.2 | ↓32.9 | 34.2 | 36.8↑ | 3.9 |
| 9 | Sputum | The number of colony | ↓35.7 | 36.2 | 35.9 | 36.4 | 38.8↑ | 3.1 |
| 10 | Sputum | DNA positive | 35.4 | 34.8 | ↓34.6 | 36.1 | 36.9↑ | 2.3 |
| 11 | Sputum | 1+ | 34.9 | ↓34.6 | 34.7 | 35.6 | 36.6↑ | 2.0 |
| 12 | Sputum | The number of colony | 35.5 | 35.7 | ↓34.7 | 36.2 | 37.4↑ | 2.7 |
| 13 | Sputum | DNA positive | 38.4 | 39.9↑ | 37.2 | ↓0.0 | 0.0 | > 30 |
| 14 | Sputum | DNA positive | 32.2 | 33.1 | 33.2 | 34.0↑ | ↓0.0 | > 30 |
| 15 | Sputum | DNA positive | 28.2↑ | 38.0 | 37.2 | 40.0 | ↓0.0 | > 30 |
| 16 | Sputum | DNA positive | ↓35.3 | 35.3 | 35.5 | 37.2 | 38.0↑ | 2.7 |
| 17 | Sputum | DNA positive | 35.9 | 35.3 | ↓34.7 | 37.1 | 37.4↑ | 2.7 |
| BALF: bronchoalveolar lavage fluid; Ct: threshold cycle; DNA: deoxyribonucleic acid; RIF: rifampin; SBL: sputum bacillary load | | | | | | | | |

**Supplementary figure S1.** Bi-directional influence of Ct value on Xpert^®^ detection results


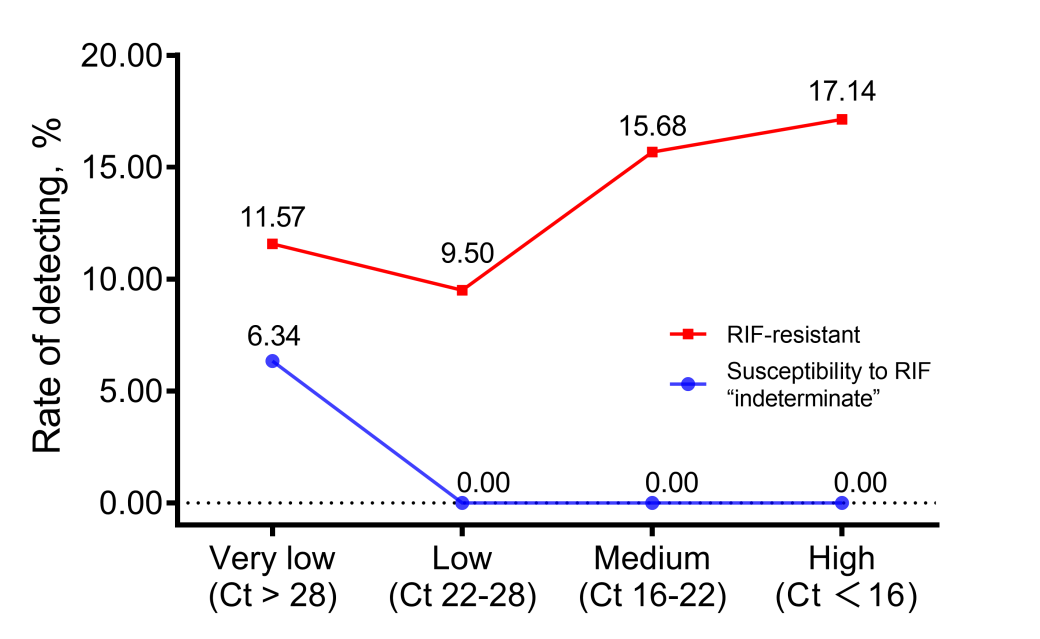


**Supplementary figure S2.** Receiver operating characteristic curve analysis of Ct value for distinguishing RIF resistance detection


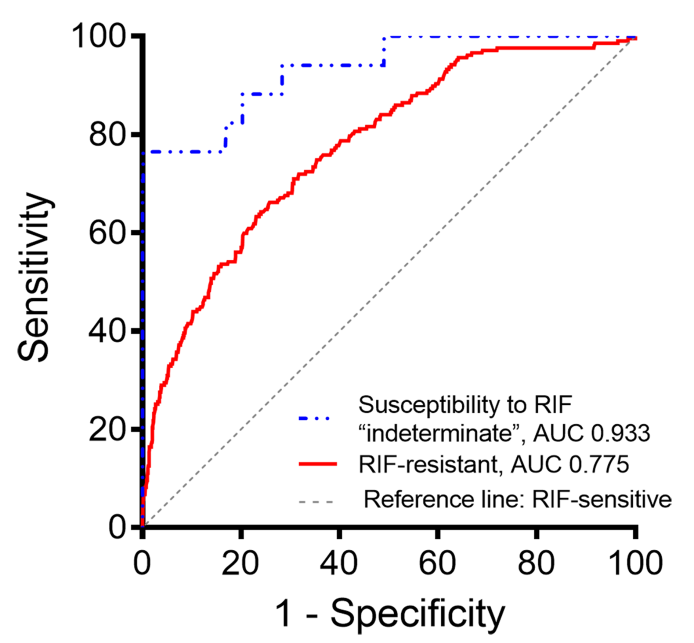

Supplement: Supplementary file 1 — Additional file 1: Supplementary table S1. Operation steps of detection method. Supplementary table S2. Sputum bacillary load report standard. Supplementary table S3. Comparison of the positive detection rates between combination of different methods with Xpert®. Supplementary table S4. Comparison of three methods in the diagnosis of bacteriologically confirmed pulmonary TB. Supplementary table S5. Correlation between sputum bacillary load and Ct value of GeneXpert®. Supplementary table S6. The distribution of rpoB mutations detected by Xpert® (n = 207). Supplementary table S7. Detection of Ct level and RIF resistance in Mtb-positive clinical specimens using Xpert®. Supplementary table S8. Comparison of the positive rate of Xpert® between initial treatment and retreatment. Supplementary table S9. Positive detection rate of Xpert® in different bacterial status of sputum (n = 980). Supplementary table S10. Clinical and laboratory characteristics of 17 cases with “indeterminate” result for susceptibility to RIF. Supplementary figure S1. Bi-directional influence of Ct value on Xpert® detection results. Supplementary figure S2. Receiver operating characteristic curve analysis of Ct value for distinguishing RIF resistance detection. [file 12879_2023_8832_MOESM1_ESM.docx]
